# The impact of a home visiting program on the care environment of Brazilian adolescent mothers - an descriptive exploratory study

**DOI:** 10.3389/fgwh.2025.1530351

**Published:** 2025-04-25

**Authors:** Letícia Aparecida da Silva, Luciola Demery Siqueira, Larayne Gallo Farias Oliveira, Euripedes Constantino Miguel Filho, Guilherme Vanoni Polanczyk, Lislaine Aparecida Fracolli

**Affiliations:** ^1^ Department of Public Health, School of Nursing, University of São Paulo, São Paulo, Brazil; ^2^ Department of Public Health Nursing, Paulista School of Nursing, Federal University of São Paulo, São Paulo, Brazil; ^3^ Institute of Psychiatry, Hospital das Clínicas FMUSP, São Paulo, Brazil

**Keywords:** parenting skills, home environment, teenage pregnancy, maternal education, child development, parenting, social vulnerability

## Abstract

**Background:**

There is evidence of a link between the environment and child development (CD) in early childhood, justifying the importance of studying the characteristics of the environment in order to understand it and thus intervene in CD.

**Objectives:**

To describe the changes in the environment of families who participated in the Young Caring Mothers Programme (YCMP).

**Method:**

This is an exploratory, descriptive study focusing on the home environment of adolescent mothers and their babies supported by the YCMP, derived from the randomised controlled clinical trial “The effect of the Young Pregnant Women Visitation Program on child development: a pilot study” (registered at clinicaltrial.gov; identifier: NCT02807818).

**Sample:**

80 pregnant adolescents, 40 in the intervention group (IG) and 40 in the control group (CG).

**Instrument:**

IT-HOME inventory.

**Results:**

At 6 and 12 months, both groups showed a tendency for the median to increase, although this increase was more pronounced in the IG. From 12 to 24 months, both groups showed a tendency for the median to decrease, with a more marked decrease in the CG, which reached values lower than those previously observed. No significant differences were found between the groups in the overall IT-HOME scores, but it was found that the relationship between maternal schooling and the score on the subscales emotional and verbal responsibility of the caregiver was greater in the control group (4. 5 points more) in mothers with less schooling (primary school) than in mothers with the same schooling in the control group (*p* = 0.02), this satisfactory result was obtained in the 6 and 24 month measurements, in the latter the intervention group scored 3 points higher than the control group (*p* = 0.05).

**Discussion:**

The results show a small impact of the YCMP on the quality of the “environment” of the families supported, but the impact is on a dimension of the environment that is very important for child development: responsive relationships of the mother with low schooling and high vulnerability.

**Conclusions:**

The YCMP can have an impact on the care environment of children under 3 years of age in families with high social vulnerability.

## Introduction

For several years, the environment to which children are exposed, particularly the home environment, has played a central role in studies of child development (CD). There is evidence linking environmental factors to CD, particularly during early childhood. This evidence suggests that the more “stimulating” the environment, the better the child's development (1, 2). Consequently, it is essential to invest in public policies that provide parents and caregivers with the knowledge and tools to transform their homes and family dynamics into stimulating environments characterized by regular and reciprocal affective interactions with the child, thus capitalizing on the extended time they spend together (1, 3).

On the other hand, a “stimulus-poor” environment, an “unfavorable environment”, has a negative impact on the rhythm of CD because it makes the child's interaction with the physical environment (home and neighborhood) and the social environment (relationships with the mother and family/caregivers) of poor quality. Poor social, emotional and environmental interactions limit the child's ability to learn (3). Approximately 69% of children living in the city of São Paulo (Brazil) do not have access to early childhood education, leaving them dependent on their parents and caregivers for their learning and development (4).

Studies confirm that a family environment conducive to CD is a protective factor against the physical and psychosocial adversities that vulnerable children may face (5, 6). Among these adversities, adolescent motherhood, for example, is often associated with adverse circumstances, such as low socioeconomic and educational status and single parenthood, which affect the quality of environment and interaction that these mothers provide for their children, resulting in cognitive and language deficits (7, 8). A stimulating environment not only ensures good CD, but also protects against the physical and psychosocial adversities that socioeconomically disadvantaged children may face (5, 6). Even in the face of financial deprivation and/or lack of access to early childhood centers, a stimulating home environment compensates and allows children to develop their potential.

In response to this scenario, the Young Caregiving Mothers Program (YCMP) was developed by researchers at the University of São Paulo as an intervention initiative aimed at promoting child development (CD). The project, implemented in São Paulo, Brazil, aims to promote positive parenting practices to enhance CD, targeting adolescent mothers living in socially vulnerable conditions.

This program uses soft technologies as part of an innovative care initiative in Brazil, that aims to promote parenting skills to create a stimulating and protective environment for children. Demonstrating its potential to cultivate positive parenting practices - reflected in an environment conducive to child development (CD) - could pave the way for its integration into public policies targeting early childhood development (9).

The YCMP aims to promote early childhood health and development through home visiting in the following ways: (1) improve maternal and infant health outcomes; (2) promote sensitive and responsive nurturing relationships between mothers and infants; (3) develop positive parenting in the mother (or the mother's ability to be attentive to the child's physical and emotional needs and to take sustained action to meet those needs); (4) stimulating cognitive and emotional interactions between mothers and infants (attachment); (5) working together to improve and strengthen the family and social support network; and (6) supporting interventions to improve adolescent mothers' self-efficacy (defined as an individual's ability to achieve a better fit between work and life) (9).

To this end, the program promotes an intervention of home visits (HV) carried out by nurses, starting between 8 and 16 weeks of pregnancy and ending at 24 months of the child's life or at any time if the adolescent requests its interruption. The frequency of HV can be bi-weekly (during pregnancy and from 2 to 20 months of the child's life), weekly (at the beginning of pregnancy and during the puerperium) or monthly (from 21 to 24 months of the child's life).

The study, The Effect of the Young Pregnant Women's Home Visiting Program on Child Development: A Pilot Study, a randomized controlled trial (RCT) conducted between August 2015 and May 2018, served to evaluate the effectiveness of the YCMP. The study was approved by the ethics committees of the Faculty of Medicine of the University of São Paulo, the University Hospital of the University of São Paulo, and the Municipal Health Department of São Paulo, and adhered to ethical guidelines, with all participants providing informed consent. The study was also registered at ClinicalTrials.gov (identifier: NCT02807818).

As part of such research, the present article aims to highlight show changes in the environment of adolescent mothers who participated in the Young Caring Mothers Program (YCMP), using the Infant-Toddler version of the Home Observation Measurement of the Environment Inventory (I-HOME) (10).

## Methodology

This is an exploratory, descriptive study focused on the home environment of adolescent mothers and their babies accompanied by the Young Caring Mothers Program (YCMP), which took place in the city of São Paulo. The research database “The effect of the Visitation Program for Young Pregnant Women on child development: a pilot study” (registered at clinicaltrial.gov; identifier: NCT02807818) was used in this study.

Participants were identified at the Primary Health Care (PHC) in the western region of São Paulo city after the start of prenatal care. At this stage, adolescents became potential participants and were given a flyer with a brief explanation of the study. With the pregnant women's consent, their contact information was added a list provided by the PHC to the research manager. The research manager then contacted the pregnant women and invited them to an interview at the YCMP headquarters. All participants who met the inclusion criteria and expressed interest in the study signed an informed consent form (ICF), which was approved by the Institutional Review Boards of the Faculty of Medicine of the University of São Paulo (ref: 052/15) and the Municipal Health Department of São Paulo. For those who were minors, the informed consent form was provided and their legal guardians were contacted to sign the form.

This study is part of a clinical trial that using convenience sampling. The intended outcome of the clinical trial was to promote better brain development, as well as improve cognitive, language and motor development milestones, and improve the quality of stimulation and support available to the child in the home environment. This last outcome will be analyzed here.

A total of 169 pregnant adolescents participated in the study, classified according to the inclusion criteria: (a) aged between 14 and 19 years, (b) primiparous, (c) gestation between 8 and 16 weeks, (d) low social class (classes C, D, E according to the ABEP scale), (e) living in the West of São Paulo11. Of these 169 pregnant women, 80 were included in the study and randomized into two groups: the intervention group and the control group. The intervention group (*n* = 40) received DV from the YCMP in addition to their usual care in the public health network, while the control group (*n* = 40) received only their usual care in the public health network. To ensure homogeneous groups, randomization was stratified according to the type of PHC that serves as the reference for the care of pregnant women, but also according to the educational level of the mother of the pregnant adolescent.

In the intervention group, participants received home visits from nurses who had completed a 40-h theoretical and practical training program. The nurses followed a standardized visitation protocol that organized their intervention around three primary theoretical frameworks: attachment theory, self-efficacy theory, and the bioecological model. The protocol included a total of 60–63 home visits, divided into four distinct phases: 14–17 visits at biweekly intervals during pregnancy; 6 visits at weekly intervals during the postpartum period; 36 visits at biweekly intervals from 3 to 20 months of infant age; and 4 visits at monthly intervals from 21 to 24 months of infant age. During follow-up, some of the participants in both the control and intervention groups moved out of the study area, resulting in a loss of participants.

All participants underwent outcome assessments conducted by interviewers blind to the mothers' participation status. Assessments were conducted at the time of program entry (baseline), then at 30 weeks of gestation, and at 3, 6, 12, and 24 months of the child's life as illustrated in the consort provided as Supplementary Material. These assessments focused on general maternal health, mental and environmental health, exposure to stress, social support, child neurodevelopment, and exposure to neglect and abuse. The study used the Home Observation for Measurement of the Environment Inventory (IT-HOME version) and a researcher-developed sociodemographic questionnaire that included questions about marital status, education, declared skin color, occupation, income, use of social services, and housing characteristics.

The independent variables were collected at the baseline interview. The baseline sample characterization included information on maternal age, declared skin color (ethnicity), school enrollment, educational attainment, occupation, grandmother's education, family participation in welfare program, the number of persons living in the household, and total monthly family income.

The dependent variable in this study is the quality of the home environment as assessed by the Home Observation for Measurement of the Environment (HOME). HOME is a 45-item instrument designed to assess the caregiving environment in which a child is raised. It has been translated into Brazilian Portuguese and has used in different regions and contexts throughout the country. Data are collected through direct observation of the home environment and through interviews with the primary caregiver. Scores are categorized into six domains (also called sub-scales), each with a maximum score: Emotional and verbal responsiveness of the primary caregiver (11 points); Absence of restriction and punishment (8 points); Organization of the physical and temporal environment (6 points); Availability of materials, toys, and games appropriate for the child (9 points); Involvement of the primary caregiver with the child (6 points); and Opportunity for variation in daily stimulation (5 points). The total IT-HOME score is calculated by summing the scores from each subscale, with a maximum possible score of 45 points. The IT-HOME score provides a profile of the family regarding the level of stimulation in the child's environment. Consequently, the following classification was established: a score of 0–25 points is considered to be below the fourth quartile, a score of 25–36 points considered to be intermediate, and a score of 37–45 points is considered to be above the fourth quartile, as developed by Caldwell and Bradley (1984). For the analyses of this study, scores between 0 and 24 are considered to indicate low environmental quality, scores between 25 and 36 are considered to indicate acceptable environmental quality, and scores between 37 and 45 are considered to reflect a good environmental quality.

A descriptive and comparative analysis was performed based on the IT-HOME information, calculating the total score and the subscales of the IT-HOME inventory for the intervention (IG) and control (CG) groups. Sociodemographic data such as mother's education and presence of a partner were evaluated to check their influence on the comparison of some of the IT-HOME inventory scores between CG and IG. The data treatment did not consider variables with incomplete data, only the variable of schooling was treated when there was an inconsistency. The nonparametric statistical tests used (Wilcoxon Rank Sum Test - most appropriate for the analysis of a small sample) removed the rows with incomplete data from the analysis. The statistical tests were applied according to the needs and indications of the professional statistician who supported the study, using a 5% significance level.

## Results

**Figure 1:**
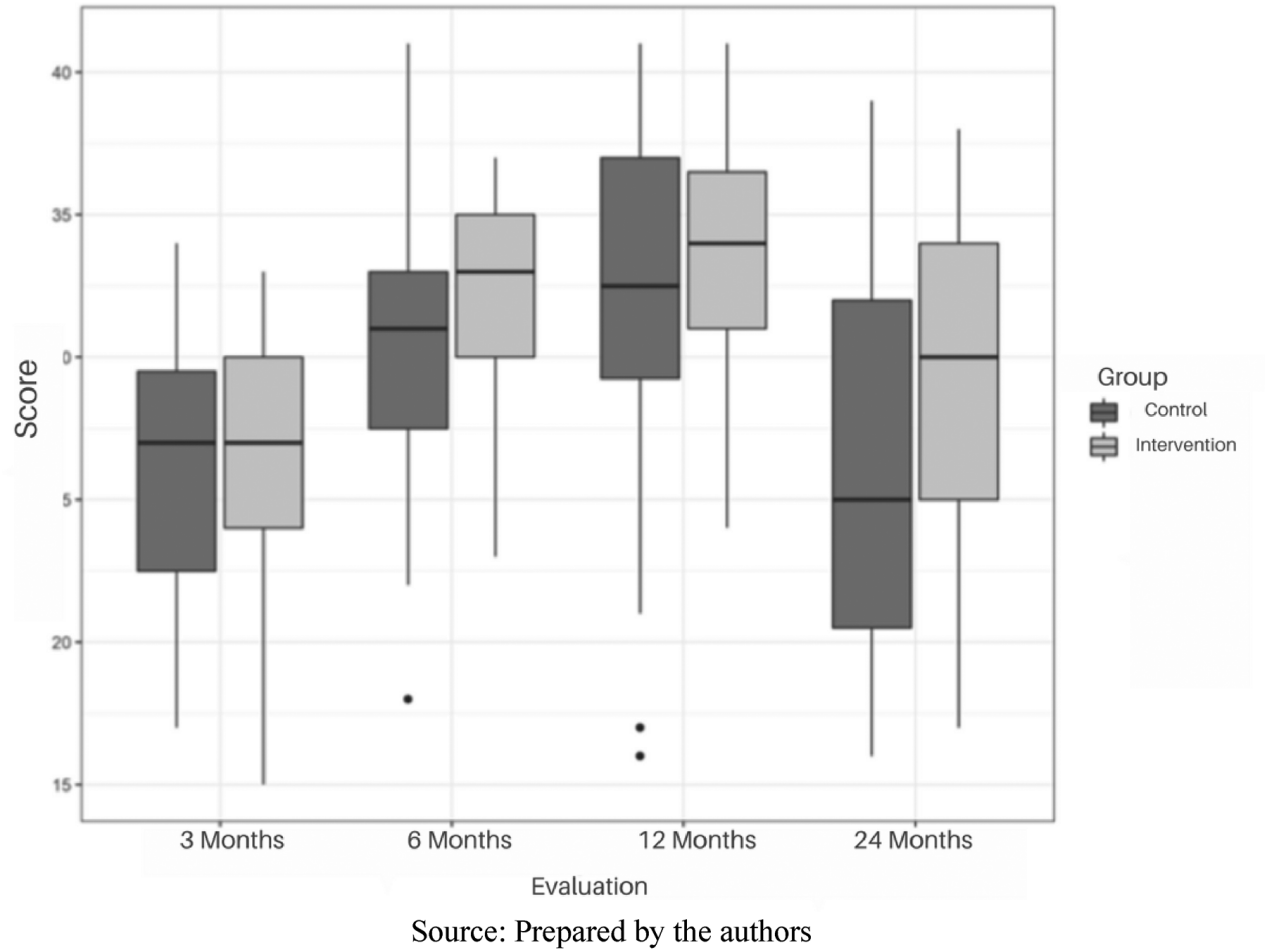
Analysis of IT-HOME total scores of the control and intervention groups over time. São Paulo 2024. Prepared by the authors.

According to the analyses, both the GC and IG showed different median scores at all assessment points, except at 3 months, where the median scores of both groups were identical. At 6 and 12 months, both groups showed a tendency for the median to increase, although this increase was more pronounced in the IG. From 12 to 24 months, both groups showed a tendency for the median to decrease, with a more pronounced decrease in the GC, which reached values lower than those observed at the 3-month assessment. The median IT-HOME total score at 24 months was 25 for the GC and 30 for the IG. The median difference between the groups varied over time, with the largest difference being 5 points at 24 months. Figure 1 illustrates the distribution of the total IT-HOME scores for both GC and IG at each time point at which the instrument was administered.

In analyzing the relationship between maternal schooling and the family's score on the IT-HOME Inventory, it was observed that changes in the total IT-HOME score followed similar patterns between the CG and IG, particularly when considering the scores of mothers with primary education. The main difference was that the IG started with higher scores compared to the CG, and these scores remained higher over time, although both groups showed a decline in scores at the 24-month assessment. In particular, for the CG, the decline at 24 months resulted in a score lower than the baseline.

**Figure 2:**
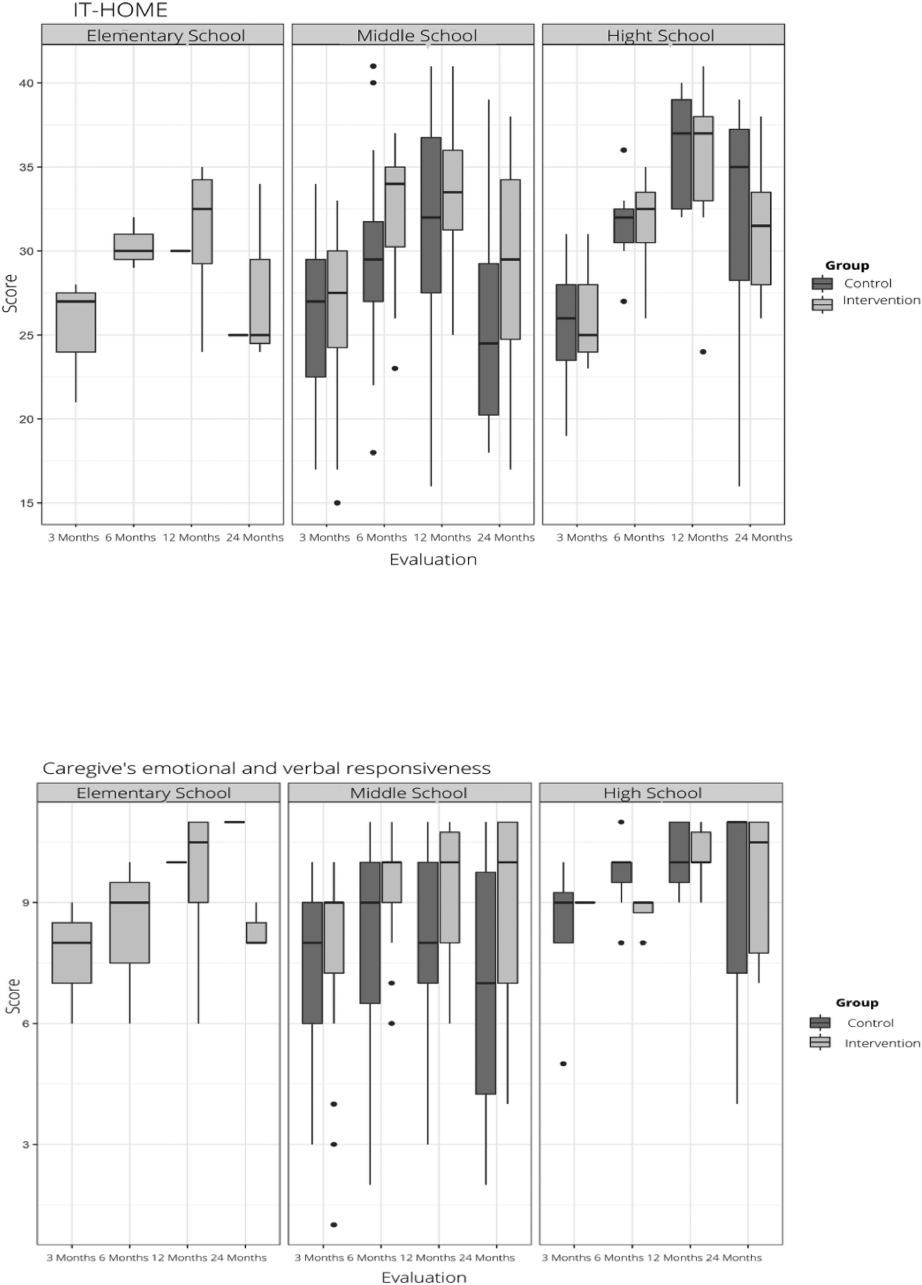
Relationship between maternal schooling and total IT-HOME score and subscores for IG and CG over time. São Paulo, 2024. Prepared by the authors.

However, statistically significant data were found for mothers with an elementary school education at the 6-month assessment time point in the total score, when the IG scored 4.5 points higher than the CG (*p* = 0.02); and in the Emotional and Verbal Responsibility of the Caregiver subscale, when the IG scored 1 point higher than the CG (*p* = 0.01). In this subscale, we also found statistically significant data for the same level of schooling at 24 months, when the IG scored 3 points higher than the CG (*p* = 0.05). These results are presented in Figure 2 below:

## Discussion

The results of this study show a small impact of the YCMP on the quality of the family environment. However, the results are promising as they show a qualitative impact on the childcare environment, especially on maternal responsiveness. The literature shows that adolescent mothers with low levels of schooling have many difficulties in establishing affective bonds and caring for their babies, and the YMP proved to be capable of helping in this area.

Recent studies have shown a mediation between maternal schooling and child development factors (11–13), so it is very important to study the schooling of caregivers is studied and relate it to the quality of the environment in which Brazilian children live and develop.

In analyzing maternal education, it can be assumed that in the general population, mothers with more education have been exposed to a greater amount of information about child development and are therefore able to provide higher quality care, provide more appropriate and varied stimulation, establish healthier relationships from a physical and emotional standpoint, and expose their babies to fewer risks. Maternal education influences child development through factors such as parenting practices, organization of the home environment, experience with stimulation materials, and others (14).

Analyzing the IT-HOME total score graphs, it can be seen that the YCMP promotes better outcomes for mothers with less schooling, as there was a greater difference in scores between groups for those with primary schooling. Mothers in the IG with primary education scored higher than mothers in the intervention group with high school education at the child's 3- and 6-month assessments. In addition, there was statistical significance at 6 months. This may also indicate that the intervention had more effective results up to 6 months after the baby's birth, as the intervention enabled mothers with low levels of education to provide a higher quality environment for the child compared to a caregiver with higher levels of education.

The mother's level of education is considered to be an aspect that can influence child care in the home environment. This aspect can be a mediator of the subscales caregiver's emotional and verbal responsiveness, caregiver's involvement with the child, and opportunity for variation in daily stimulation (1, 15). Analyzing the subscales, it can be seen that the caregiver's emotional and verbal responsiveness and the caregiver's involvement with the child showed the greatest difference between the groups when the mother's education was primary school. This is in line with the expectations of the YCMP, since the visiting nurses talked about the importance of communicating with the child, considering them as a subject with desires and needs, recognizing and praising their skill gains with affection, so that even mothers with less schooling could understand and apply the nurse's guidance.

In the YCMP sample, schooling may have a statistically significant relationship with performance on the IT-HOME Inventory only at 6 months for the total score and at 6 and 24 months for the caregiver emotional and verbal responsibility subscores. In a previous study analyzing the relationship between the quality of home stimulation and the cognitive performance of children aged 17–42 months, the authors (16) identified the influence of maternal education on the quality of this stimulation by examining the children's schooling. This study (16) found an average total IT-HOME score of 27.9 for mothers with more than 5 years of schooling (equivalent to primary school or more), a result that was lower than that found for all measurement times (within the same age range) in the YCMP sample. In the sample from the previous study mentioned above, only the absence of restriction and punishment behaved independently of schooling.

Contrary to the results of another study carried out in São Luís - MA (17), which aimed to analyze the home environment of 176, 2-year-old children in terms of stimulation and its relationship with suspected developmental delays in a low-income suburban community, the authors showed that low parental schooling (less than or equal to 5 years of schooling - equivalent to the period from primary to secondary school) had a significant association with low IT-HOME scores.

Another study of 630 children in Pelotas (RS) (18) sought to identify risk factors that might be associated with environmental quality and found a strong association between maternal schooling and environmental quality. Although this study used the MC-HOME version for preschool children, it is worth mentioning here to illustrate that the association between environmental quality and maternal schooling is relevant throughout childhood.

The IT-HOME Inventory measures the quality of the environment, taking into account: quality of care, level of stimulation, and exposure to risks or limiting elements. Therefore, the results of this research, presented above in the IT-HOME total score, show that the intervention was able to raise the performance of mothers with less schooling to a level of mothers with more schooling, up to 12 months of the child's life.

## Conclusion

This article shows the results of the IT-HOME scale in terms of maternal schooling in the IT-HOME total score and in the subscales, while other studies show results only in terms of the IT-HOME total score.

The results of this study show a small impact of the YCMP on the quality of the family environment. However, the results are promising as they show a qualitative impact on the childcare environment, especially on maternal responsiveness. The literature shows that adolescent mothers with low levels of schooling have many difficulties in establishing affective bonds and caring for their babies, and the YMP proved to be capable of helping in this area.

The results presented above show that supporting the construction of quality environments, despite the unfavorable social conditions can produce beneficial results for CD. For this, it is necessary to invest in the identification of protective factors for parenting care, especially in scenarios of greater vulnerability, as is the case in most of Brazil.

In terms of implications for nursing practice, this study contributes to alerting managers and professionals to the need to structure nursing interventions aimed at building favorable home environments for caring for children aged 0–3 years. The results showed changes in maternal responsiveness and care, despite the mother's vulnerability. To do this, nurses may need to step out of their usual “therapeutic setting” and expand their comfort zone to deeply understand the reality and be more empathetic when caring for vulnerable families with young children.

Currently, the YCMP is called the First Ties Program and is being implemented as a public policy in the municipalities of Jaguariúna and Indaiatuba in São Paulo State (REF). To facilitate this implementation, several adaptations have been made, including a reduction in the number of visits and the involvement of nurses who are already part of the municipal health network. It is hoped that these modifications will increase acceptance, improve adherence, and improve both distribution and integration into the general public health system.

To contribute beyond the confines of primary care, this study recommends the inclusion of parenting as a topic in nursing school curricula. While nursing education equips nurses to be recognized as key figures in the development of parenting (19), this topic is typically addressed only in specific courses focused on child care. If parenting were more broadly integrated into the undergraduate curriculum, nurses would be better equipped to implement strategies that promote parenting in all areas of nursing practice.

A limitation of the study is that the YCMP was an intervention within an RCT funded by research grants from funding agencies, it was difficult to retain professionals during the study and the frequently change of visiting nurse may have had an impact on the quality of the intervention provided by the YCMP. The reason is, despite theoretical and technical training, the experience gained in delivering the Home visits is a substrate that cannot be provided by training and is related to the quality of the intervention. Another limitation is the small sample size and the loss of participants over time.

## Data Availability

The datasets presented in this study can be found in online repositories. The names of the repository/repositories and accession number(s) can be found below: registrado em clinicaltrial.gov/identificador: NCT02807818.
