# Supplementary figures and images for: The impact of a home visiting program on the care environment of Brazilian adolescent mothers - an descriptive exploratory study
Source: Front Glob Womens Health. 2025 Apr 25;6:1530351. doi: 10.3389/fgwh.2025.1530351 (PMC12061960; doi:10.3389/fgwh.2025.1530351)

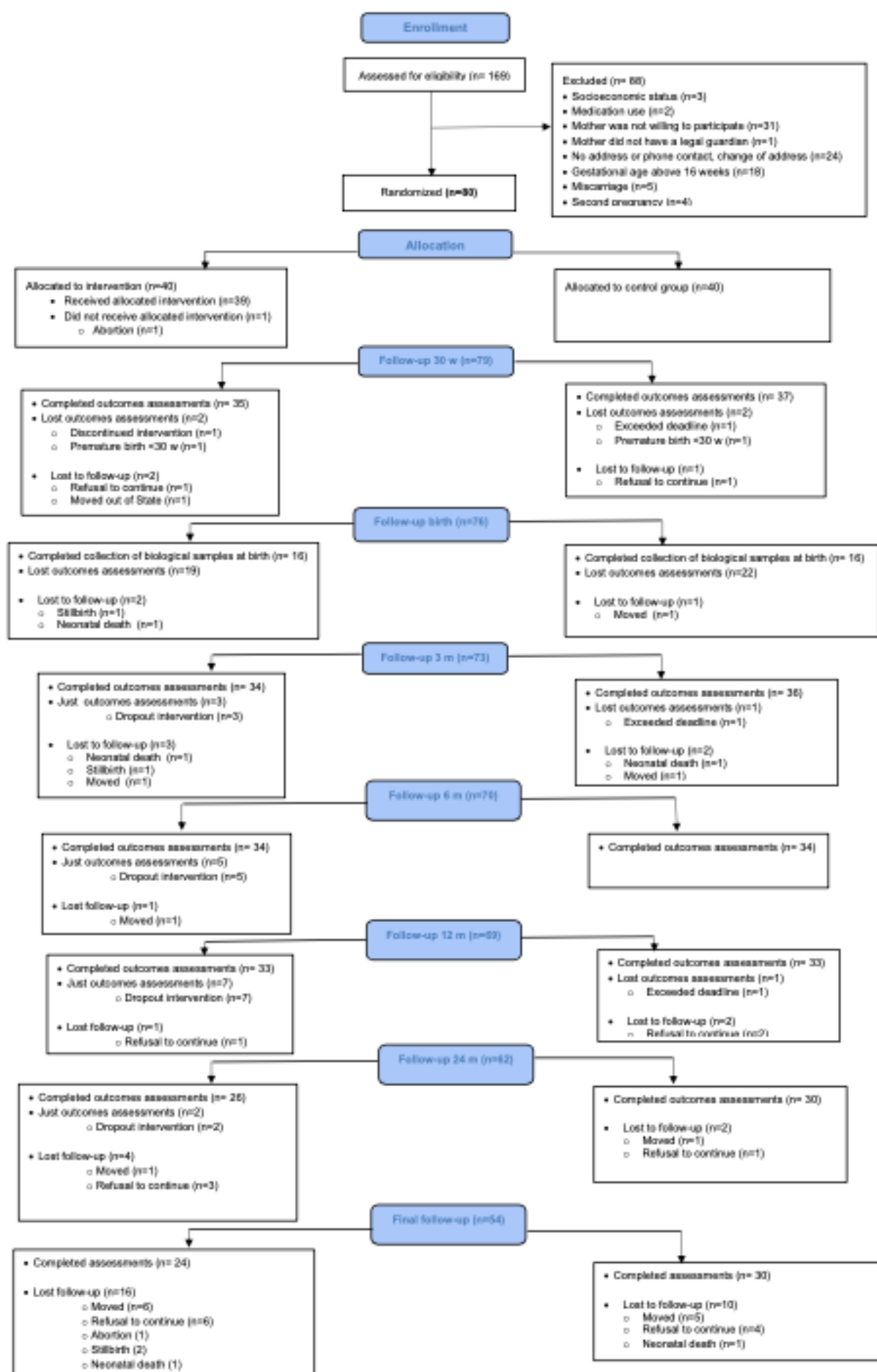

Supplement: Supplementary file 1 [file Datasheet1.pdf]
